# Best Practices in the Development, Translation, and Cultural Adaptation of Patient-Reported Outcome Measures for Adults With Hearing Impairment: Lessons From the Cochlear Implant Quality of Life Instruments

**DOI:** 10.3389/fnins.2021.718416

**Published:** 2021-11-24

**Authors:** Ariane Laplante-Lévesque, Judy R. Dubno, Isabelle Mosnier, Evelyne Ferrary, Theodore R. McRackan

**Affiliations:** ^1^Department of Clinical Evidence Cochlear Implants, Oticon Medical A/S, Smørum, Denmark; ^2^Department of Behavioural Sciences and Learning, Linköping University, Linköping, Sweden; ^3^Department of Otolaryngology-Head and Neck Surgery, Medical University of South Carolina, Charleston, SC, United States; ^4^Hearing Institute, Institut Pasteur/Université de Paris/Inserm, Paris, France; ^5^Unité Fonctionnelle Implants Auditifs, ORL, GH Pitié-Salpêtrière, AP-HP Sorbonne Université, Paris, France

**Keywords:** patient-reported outcome measure (PROM), questionnaire, PROM development, PROM translation, PROM cultural adaptation, quality of life, hearing impairment, cochlear implant

## Abstract

This manuscript summarizes available evidence-based best practices in the development, translation, and cultural adaptation of one type of outcome measure for adults with hearing impairment, patient-reported outcome measures (PROMs). It presents the development of the Cochlear Implant Quality of Life (CIQOL) instruments and the ongoing translation and cultural adaptation of the CIQOL-35 Profile from English to French as case studies and discusses useful lessons for selecting, developing, translating, culturally adapting, and using PROMs. Relevant best practice guides are introduced, described and their steps are illustrated with examples. Future trends in hearing-related PROMs, including computerized adaptive testing, patient-reported experience measures (PREMs), economic evaluation and allocation of scarce resources, and PROMs in low-resource settings, are discussed. The manuscript concludes on the lessons that can be learned from implementation science for the successful and sustainable integration of PROMs in clinical practice.

## Introduction

This manuscript, part of the Research Topic “Outcome Measures to Assess the Benefit of Interventions for Adults with Hearing Loss: From Research to Clinical Application,” summarizes already available evidence-based best practices in the development, translation, and cultural adaptation of patient-reported outcome measures (PROMs) for adults with hearing impairment. It presents the development of the Cochlear Implant Quality of Life (CIQOL) instruments and the ongoing translation and cultural adaptation of the CIQOL-35 Profile from English to French as illustrative case studies for those interested in selecting, developing, translating, culturally adapting, and using PROMs.

### Hearing Impairment and Patient-Reported Outcome Measures

Hearing loss is the most prevalent sensory disorder and the third most common cause of Years Lived with Disability (YLDs) after low back pain and migraine, in the 2019 Global Burden of Disease (GBD) study, a systematic overview of the prevalence of 369 diseases and injuries (Haile et al., 2021). The World Hearing Organization urges for multi-disciplinary hearing health care action including prevention and rehabilitation. The GBD study defines hearing loss as a pure-tone average of audiometric thresholds at 0.5, 1, 2, and 4 kHz ≥ 20 dB HL in the better ear. This definition focuses only on hearing detection/acuity and, therefore, does not consider functional abilities, self-reported hearing difficulties, or their impact on quality of life. Globally, more than 1.5 billion people, or 20% of the population, experience some degree of hearing loss (World Health Organization [WHO], 2021b).

Hearing evaluation is mostly performed through pure-tone thresholds measurements, speech recognition tests, and other standard diagnostic assessments designed to differentiate conductive and sensorineural hearing loss, and less-so on PROMs (Granberg et al., 2014; Hill-Feltham et al., 2020). A systematic review reported that PROMs represent only 9% of the total hearing outcome measures (*n* = 837), whereas pure-tone thresholds measurements and speech recognition tests accounted for 65 and 20%, respectively (Hill-Feltham et al., 2020).

Quality of life refers to an “individual’s perception of their position in life in the context of the culture and value systems in which they live and in relation to their goals, expectations, standards and concerns” (World Health Organization [WHO], 2021a). Health-related quality of life focuses on the aspects of quality of life most relevant for health, i.e., the physical, mental and social well-being (Guyatt et al., 1993). To evaluate the impact of hearing loss on quality of life or the benefit of hearing-related interventions on quality of life, numerous PROMs have been developed, including hearing-specific instruments, hearing-aid-specific instruments, and cochlear-implant-specific (CI) instruments (Andries et al., 2021). Health-related quality of life measures, such as the Euro-QoL (EQ-5D), the Health Utilities Index (HUI3), and the SF-36, are often used to measure health utility, but are weakly associated with hearing-specific PROMs due to the lack of items that are related to everyday functional communication and social interaction (McRackan et al., 2019a,^2021^).

In contrast to standard audiometric test batteries, a direct input assessment from patients of improvement of their health and quality of life following CI is recommended to evaluate the positive and multidimensional impact of hearing rehabilitation. PROMs/quality of life measures are increasingly being regarded as quality indicators. For example, in England the National Health Service has a National PROMs Program under which it coordinates the national collection of PROMs for four elective surgical procedures (National Health Service [NHS], 2021). In the United States, the Meaningful Measures framework identifies priority areas that promote quality healthcare for Medicare and Medicaid patients and include functional outcomes and patient experiences of care feature (Centers for Medicare and Medicaid Services [CMMS], 2021). Moreover, PROMs/quality of life measures are now mandatory in some countries for reimbursement of medical devices and requested to identify most personalized care pathways (Patrick et al., 2007; Artières-Sterkers et al., 2020; Fraysse et al., 2020). For example, the U.S. Food and Drug Administration positions appropriate PROMs as central to clinical studies that evaluate the effectiveness of new medical products including hearing devices (Patrick et al., 2007).

### Currently Available Patient-Reported Outcome Measures

A synthesis of available PROMs identified that they target the following three domains: auditory (listening, communicating, and speaking), social (relationships, isolation, social life, occupational, and interventions), and self (effort and fatigue, emotions, identity, and stigma; Vas et al., 2017). However, limited evidence is available to support the unidimensionality of these domains. For example, recent re-evaluation of the Hearing Handicap Inventory for the Elderly did not show the social and emotional domains to be independent (Cassarly et al., 2020). Indeed, some of the legacy measures sometimes have unknown or unsound psychometric properties, including face/content validity. It is common for researchers and clinicians to have developed PROM items without relying on expert panels of patients, focusing instead on input from clinicians. This means the domains/items included may not cover the issues most important to the patient population. It is recommended to include qualitative research methods and literature reviews in the development of outcome measures, as detailed in the next sections. Another limitation of some legacy PROMs is that they are not always efficient, with some domains including more items than necessary, which creates burden for the patient, the clinician and the researcher, and reduces the likelihood that the PROM will be used in routine clinical practice and clinical research.

### Development of Patient-Reported Outcome Measures–Cochlear Implant Quality of Life

Modern development standards for PROMs aim to create efficient, precise, and responsive instruments that represent the values most important to the population of interest. The Patient-Reported Outcomes Measurement Information System (PROMIS) and COnsensus-based Standards for the selection of health status Measurement INstruments (COSMIN) have established standards that aim to improve the quality of PROMs used to measure clinical and research outcomes (Mokkink et al., 2010; PROMIS, 2013). While differences exist, both support the use of a mixed methods research design and agree on an overall structure. We illustrate this process through a case study, the development of the CIQOL-35 Profile instrument and CIQOL-10 Global measure.

#### Systematic Literature Review

A systematic literature review is necessary for step one in the PROM development process in order to develop a comprehensive understanding of the previous work done in the area of interest. In addition, the previous items and concepts included in legacy instruments can help form the protocols for future focus groups or key informant interviews. As a part of the CIQOL development process, two systematic reviews and meta-analyses identified 21 studies that used pre- and post-implantation PROMs to monitor adult CI outcomes (McRackan et al., 2018a,b). Overall, this identified a clear improvement in QOL using both hearing- and CI-specific instruments and general-health QOL instruments. However, hearing- and CI-specific instruments demonstrated substantially greater improvements in QOL than general health instruments (McRackan et al., 2018a,b, 2019a). In addition, this analysis found negligible to low positive correlations between speech recognition scores (words in quiet, sentences in quiet, and sentences in noise) and patient self-reported functional ability (Table 1). This relationship was maintained even when comparing communication domains in QOL instruments to speech recognition outcomes. These results are consistent with the assumption that how patients with CIs communicate in their everyday functioning is more complex than revealed by speech recognition tasks routinely used in clinical care, which further supports the use of PROMs as part of a test battery to comprehensively assess CI outcomes.

**TABLE 1:** Meta-analysis of correlations between speech recognition scores and patient self-reported functional ability.

Speech recognition scores	*r*	95% confidence intervals
**Cochlear implant-specific quality of life**		
Word recognition in quiet	0.21	0.12 to 0.30
Sentence recognition in quiet	0.24	0.08 to 0.39
Sentence recognition in noise	0.26	−0.08 to 0.54
**Hearing-specific quality of life**		
Word recognition in quiet	0.28	0.14 to 0.37
Sentence recognition in quiet	0.20	0.07 to 0.33
Sentence recognition in noise	NA	NA
**Health-related quality of life**		
Word recognition in quiet	0.33	0.19 to 0.46
Sentence recognition in quiet	0.34	0.18 to 0.48
Sentence recognition in noise	0.32	0.19 to 0.44

#### Focus Groups and Cognitive Interviews

The next step in the PROM development process is to conduct focus groups or key informant interviews to ensure the themes and topics that affect the population interest, in this case adult patients with CIs, are included in the items in the PROM. This qualitative analysis is critical as it provides the face and content validity of the instrument. For the CIQOL, adult patients with CIs with a wide range of speech recognition outcomes took part in focus groups (McRackan et al., 2017). The 23 patients were stratified into 3 focus groups based on communication abilities with their CI as measured by word scores on the consonant–vowel nucleus–consonant (CNC) test in quiet presented at 60 dB SPL (group 1: <36%; group 2: 36–66%; group 3: >66%). Analysis of the focus group transcripts identified seven themes: communication, emotion, entertainment, environmental sounds, independence, listening effort, and social. Individual focus group participant statements related to these themes were then developed into items. This generated a 101-item pool, which served as a potential source of items to include in subsequent instruments. Audiologists, physicians and hearing science researchers then carefully reviewed the items and also ensured the items were at or below a 6th grade level reading level, using the Lexile Analyzer.^1^ Item clarity was then confirmed through cognitive interviews with 20 adult patients with CIs who were not involved in the focus groups (McRackan et al., 2017). These interviews confirmed that the items were easy to read and understand, unambiguous, and culturally appropriate. No items required revision based on the cognitive interviews.

#### Psychometric Testing to Develop the Cochlear Implant Quality of Life Item Bank

One of the most recent significant changes to PROM development is the increased use of item response theory (IRT). IRT is the core of modern psychometric analyses used to develop PROMs and has several advantages over classical test theory (CTT), which was the previous standard. First, CTT is grounded on observed and true scores, which focuses on the measurement of an underlying trait—referred to as person ability or person measure. Therefore, CTT-derived instruments are sample-dependent as subjects will have higher true scores on easier tests and lower true scores on more difficult tests. In contrast, IRT-developed instruments remain sample and test independent (Prieto et al., 2003).

Second, whilst CTT focuses on test-level psychometrics, IRT focuses on item-level psychometrics. IRT analyses concentrate on each individual item and determine its measurement characteristics and utility for inclusion in subsequent instruments. IRT analyses not only evaluate the ceiling and floor effects for each item, but also identify fit to the hierarchical model, matches individual item difficulty level to person ability level, and ensures that the items cover the full ability range of the patient population. Application of IRT analyses to the item pool results in the final item bank, which serves as the source for items to be used for subsequent PROMs (including short form, profile, and computerized adaptive testing (CAT) instruments, which will be discussed in a later section). With the psychometric properties established for each item, researchers can select items for each instrument based on their highest discrimination across the ability range and best match between item difficulty and patient ability. This results in optimized instruments with maximized capacity to differentiate individuals across a greater range of the latent trait—termed precision (Rose et al., 2008).

The third advantage is related to the stricter assumptions that must be met before IRT analysis is performed compared to CTT (Reeve et al., 2007). This includes (1) unidimensionality—items only contribute to one domain construct, (2) local independence—responses to each item are unrelated to responses to other items, and (3) item fit—the items must fit the IRT measurement model. Confirmatory factor analysis (CFA) is used to confirm unidimensionality and local independence. For item bank development, items are eliminated if they do not significantly contribute to the unidimensional construct captured by the other items in a domain, or if responses to an item are dependent upon responses to other items in the pool. In addition, item fit to the IRT model, such as infit and outfit, are examined to ensure that the included items sufficiently measure the construct of interest for individuals at ability levels close to and far from the item difficulty.

For the CIQOL item bank, the item pool of 101 items organized into 7 domain constructs was completed by 371 adult patients with CIs from all regions of the United States (McRackan et al., 2019c). By completing the psychometric analyses described earlier, one domain construct was found to lack unidimensionality (i.e., independence) and was removed from the item pool. All other domains were found to represent unidimensional constructs. In addition, several items were removed for being locally dependent on other items (*n* = 3) and misfitting the IRT model (*n* = 6). This resulted in the final item bank item which consisted of 81 items in 6 domains (communication, emotion, entertainment, environmental sounds, listening effort, and social).

#### Development of the Cochlear Implant Quality of Life-35 Profile Instrument and Cochlear Implant Quality of Life-10 Global Measure

The item-level psychometric analyses results were then used to guide the development of the subsequent instruments (McRackan et al., 2019b). Here, items are selected for each domain that represent the full ability continuum (based on item difficulty) and have the greatest capacity to discriminate individual patient ability. Additional IRT analyses can then be performed to ensure that the items selected fit each domain’s IRT model. The CIQOL-35 Profile was developed using this method and assesses outcomes represented in the 6 domains (McRackan et al., 2019b). A single factor CFA was then performed on the CIQOL-35 Profile to ensure it was psychometrically sound to use as a source for items in a global measure (CIQOL-10 Global). After this was confirmed, the CIQOL-10 Global was created based on the above parameters. This measure provides an overall assessment of CI-related functional outcomes but does not provide domain-specific information. Importantly, all items for the global measure are included in the profile instrument so a global score can be easily calculated from the CIQOL-35 Profile.

#### Final Validation of the Cochlear Implant Quality of Life-35 Profile and Cochlear Implant Quality of Life-10 Global

After the creation of the instrument, final validation typically includes comparison of psychometric properties of the newly developed PROMs to legacy instruments. Available guidelines are less concrete regarding the analyses needed for this final stage. In general, there are three main components to this comparison. First, construct validity determines whether each purported domain represents a unidimensional concept. This includes analysis of all domains, subdomains, and total scores. Second, convergent validity evaluates the degree to which scores from an instrument are associated with conceptually similar measures. This can range from correlation with physiological findings when available or legacy PROMs. Third, reliability determines the consistency of PROM scores across time.

To accomplish this, results from the CIQOL instruments were compared to results from the Nijmegen Cochlear Implant Questionnaire (NCIQ) and HUI3 in 334 adult patients with CIs who were not involved in previous development stages (McRackan et al., 2021). The results demonstrated that all CIQOL domains as well as the global measure had strong construct validity, strong convergent validity, and strong to very strong reliability. In contrast, 8 of the 10 NCIQ domains/subdomains as well as the NCIQ total score demonstrated poor construct validity. The remaining NCIQ subdomains (basic sound performance and activity limitation) demonstrated strong psychometric properties and test–retest reliability. Interestingly, HUI3 reliability was moderate to weak in adult patients with CIs with the weakest reliability in the hearing dimension. This is likely related to the use of “hearing aid” in several items, which may confuse patients with CIs.

The final product of this process are two instruments that represent the values of adult patients with CIs and are more psychometrically sound and comprehensive than previously developed PROMs. The CIQOL-35 Profile and CIQOL-10 Global are available for use in clinical and research settings and are free to download at http://education.musc.edu/CIQOL. The CIQOL instruments have been downloaded by over 210 CI centers and are undergoing translation and cross-cultural adaptation in 8 languages.

### Hearing Related Patient-Reported Outcome Measures in French Language

Most of the world’s population does not speak English, yet exchanging information beyond and across linguistic communities is crucial. PROMs developed and validated using rigorous procedures as described earlier should then be carefully translated, culturally adapted, and validated to other linguistic and cultural groups.

Currently available hearing PROMs in French language include the Abbreviated Profile of Hearing Aid Benefit (APHAB), the Client Oriented Scale of Improvement (COSI), the Hearing Implant Sound Quality Index (HISQUI), the NCIQ, and the Speech, Spatial and Qualities of Hearing Scale (SSQ). All the above-mentioned PROMs were published in English and their French translation process is undocumented. To the best knowledge of the authors, the NCIQ, which was developed in Dutch and published in English but unfortunately without a description of the translation process, is the only PROM designed specifically for patients with CIs available in French and its use in clinical practice is complex due to its length (60 items). In contrast, the Evaluation of the Impact of Hearing Loss in Adults (ERSA) was developed and validated in French and is relevant for hearing aid and adult patients with CIs. It has good reliability, validity, and sensitivity to change, but it is difficult to compare scores against other PROMs as it has yet to be translated to other languages (Ambert-Dahan et al., 2018).

The following sections describe the process of translation, cultural adaptation, and validation through a case study, the translation of the CIQOL-35 Profile instrument from English, its source language, to French. Throughout the manuscript, for clarity of expression, the term translation is used to refer to the iterative process of both translation and cross-cultural adaptation.

## Materials, Equipment and Methods

The translation of PROM items, response choices, and instructions should be obtained through an iterative process of forward and back-translation by qualified translators and bilingual content experts, bilingual expert review, and testing with the patient group. A hearing-related PROM translation good practice guide recommending six steps can be used to guide this iterative process (Hall et al., 2018b).

Materials include the source-language PROM (i.e., the CIQOL-35), good practice guides (PROMIS, 2013; Hall et al., 2018b), and a location to archive all steps and related documentation. The “reconciliation report” provided by Hall et al. (2018b) as supplemental file 3 is especially helpful in documenting the translation process.

**FIGURE 1:**
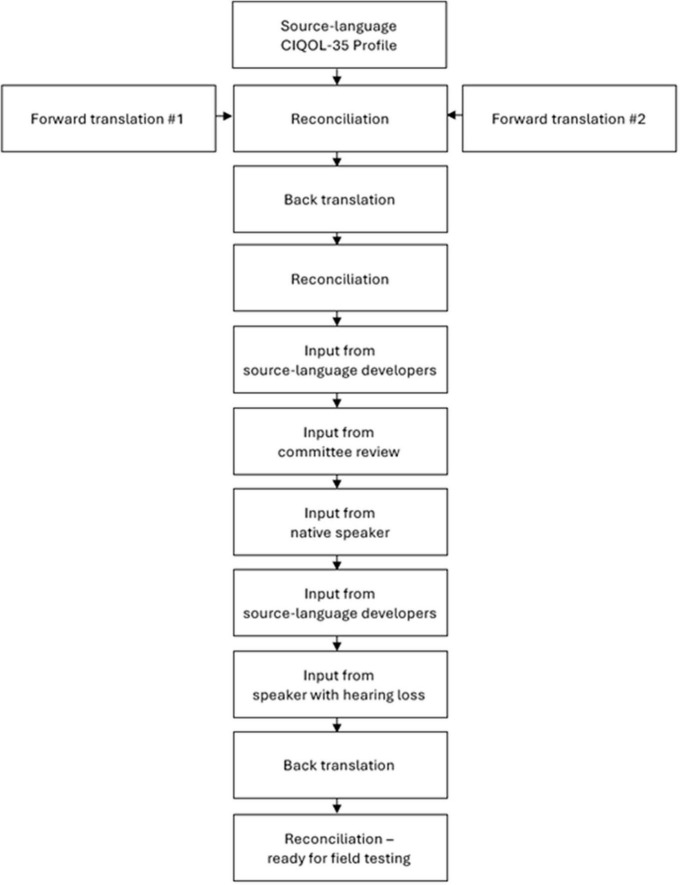
Translation and cross-cultural adaptation process of the CIQOL-35 Profile from English to French.

The following section describes the six steps of Hall et al. (2018b) and illustrates how they guide activities in the case study of the translation of the CIQOL-35 instrument from English to French. In this PROM translation project, Steps 1–4 are completed and Steps 5–6 are yet to be completed. Figure 1 summarizes the steps completed so far; these are described below.

### Step 1. Preparation

#### Summary of Best Practices

According to Hall et al. (2018b), this step sets the scene for the translation and includes checking whether a translation of the instrument already exists and gaining approval from the source-language PROM copyright holders for the translation. Source-language PROM developers should be invited to be involved as their input is important to clarify the original intent of the PROM instructions, items, and response options. The translation project should have clear aims and intended audience and the main concepts that underpin the PROM should be defined. Finally, template documents for documenting the translation should be prepared.

#### Case Study: Translation of the Cochlear Implant Quality of Life-35 Instrument From English to French

No French version of the CIQOL-35 instrument existed, as it is a recent PROM. The translation project was instigated and took form through a close collaboration between hearing clinicians and researchers who are native French speakers and the CIQOL-35 developers. The copyright holder, the Medical University of South Carolina Foundation for Research Development, provided written approval for the translation project. Aims and intended audience were discussed and agreed upon. The primary audience was similar to the source-language CIQOL-35 instrument, e.g., adults with hearing loss regardless of hearing device status. Although the CIQOL suite of instruments is designed for adult patients with CIs, it should also be valid for pre-CI measures, i.e., for adults with hearing loss before they receive one or two CIs. Efforts were made to reach all people of adult age irrespective of literacy and to create a translation that could be administered as pen-and-paper as well as electronic.

The linguistic and cultural profile of the intended audience includes French-speaking people living in France or elsewhere. French is spoken by 300 million people globally, of which 59.3% live in Africa, 33.4% in Europe, 7.0% in the Americas, and 0.3% in Asia and Oceania (Organisation Internationale de la Francophonie, 2019). The 300 million French speakers spread across the globe do not use the French language in a uniform manner. In the different geographical areas, the French language has evolved into different dialects, i.e., varieties of French that are mostly mutually intelligible, especially if they are close on the dialect continuum. This is the same phenomenon that distinguishes, for example, the English or Spanish spoken in different parts of the world. Dialects do not respect country borders and dialects can be multiple in the same country. Dialects vary in their vocabulary, grammar, and pronunciation, the latter important for example for speech perception test stimuli but not for PROMs to be administered in written form. Given the presence of dialects, two approaches to PROM translation can be taken. A specific and localized approach produces as many translations as the number of dialects. A universal approach favors a “standard” version of the language and avoids regionalisms (i.e., vocabulary words, grammatical structures, or expressions favored by speakers in a particular geographic area). For this translation, a universal approach was adopted. Universal translation runs the risk of using terms or grammatical structures that are not immediately recognized by some speakers, or that require more cognitive effort to be understood. To support a universal approach to translation, involving people familiar with different dialects, referring to linguistic resources that recognize standard and colloquial usage of terms by region (such as Joseph Wright’s English Dialect Dictionary or the Real Academia Española’s Spanish dictionary), and testing on speakers of different dialects is recommended. These considerations are also relevant during the development of a PROM and for which linguistic communities it is intended.

To prepare for the translation, a list of resources was created, which included the concept definitions used in the source-language CIQOL-35 instrument development, further concept definitions, as well as examples of hearing-related written documents available in both English and French obtained from the World Health Organization, Hear-it.org, and Oticon Medical A/S. These resources served two purposes. First, they presented background information about hearing, hearing impairment, its consequences, and its treatment. Second, they provided a range of examples of English-French translations of relevant terms. The “reconciliation report” provided by Hall et al. (2018b) as supplemental file 3 was adapted to the purposes of this translation project, including copying all CIQOL-35 instructions, items, and response options as separate spreadsheet rows and copying all supporting information and links into a separate spreadsheet within the same document, for easy access by all people involved in the translation. Table 2 lists the people involved in this translation project.

**TABLE 2:** People involved in this translation project along with their roles.

Person	Role in this translation project
Translation lead	Project management, resource management, procedure documentation/archiving, reconciliation of the translation, oversight of the field testing
Source-language PROM developers	Provision of concept definitions, consulting on queries arising during translation
Linguist #1	Forward translation including difficulty rating and participation in committee review
Native speaker health professional #1	Forward translation including difficulty rating and participation in committee review
Linguist #2	Back translation and participation in committee review
Native speaker health professional #2	Participation in committee review, also field testing investigator
Native speaker health professional #3	Participation in committee review, also field testing investigator
Native speaker reviewer #1	Revision after committee review
Native speaker reviewer #2	Revision after committee review
Linguist #3	Back translation after committee review

### Step 2. Forward Translation

#### Summary of Best Practices

According to Hall et al. (2018b), this step includes the translation of the PROM from the source language to the target language. For this, translators whose first language is the target language, and ideally, have the same dialect and reside or have lived experience of the region/culture of the intended audience should be recruited. It is recommended that at least two translators are involved, one translator that is a professional translator with training/certification in linguistics and another translator that is a healthcare professional with experience working with adult patients with CIs. The translators should be introduced to the PROM to be translated, the health condition and related concepts, as well as the concept definitions in Step 1 described earlier. They should also be instructed on the translation and adaptation task and this should be done in a uniform fashion for all translators. Each translator should work independently to produce a forward translation of the PROM instructions, items, and response options. The reconciliation of the forward translations by another person involves creating from the multiple forward translations a single forward translation.

#### Case Study: Translation of the Cochlear Implant Quality of Life-35 Instrument From English to French

The two translators recruited were both French native-speaking and had a high command of English. One was a certified linguist with experience in translation of hearing-related documents and the other held a clinical support position for Oticon A/S that includes regular contacts with patients with CIs. They received the same instructions and background information together with the spreadsheet described earlier in the previous step. They were instructed to maintain conceptual, item and semantic equivalence and that it was more important to preserve meaning than to provide a literal translation. Everyday non-technical language was to be used and a “universal translation” approach avoiding regionalisms was prioritized. As they translated each section of the PROM (i.e., each spreadsheet row), they were instructed to rate how difficult they found each translation (from 0 extremely easy to 10 extremely hard). These ratings were useful in the reconciliation step, which involves comparing and contrasting the different translations. A bilingual hearing clinician/researcher completed this task, using the same spreadsheet described earlier. The first step involved highlighting sections where the translations differed. As stated by Hall et al. (2018b), dedicated effort was spent on the sections that the translators rated as relatively more difficult to translate compared to other sections. The person completing the reconciliation documented the reasoning behind reconciliation decisions. This step resulted in one single forward translation of the CIQOL-35.

### Step 3. Back Translation

#### Summary of Best Practices

According to Hall et al. (2018b), this step involves the translation of the PROM from the target language back to the source language for comparison with the source-language PROM. The person conducting the back translation should be naive to the source language PROM. The assumption is that if the translation and adaptation process is carefully done, any differences between the source-language PROM and the back translation would reflect cultural adaptation. For this, at least one translator should be recruited, ideally a professional translator with training/certification in linguistics. The back translation is then carefully compared with the source PROM and equivalence is classified from perfect (A) to null (D) equivalence, in both choice of words and semantics conveyed, and this is recorded in the reconciliation report.

#### Case Study: Translation of the Cochlear Implant Quality of Life-35 Instrument From English to French

One certified linguist with experience in translation of hearing-related documents was recruited. This translator received the same instructions, background information and spreadsheet described in Step 2 earlier, except that the source-language CIQOL-35 instrument was not shown in the spreadsheet. The same person who completed the reconciliation task in Step 2 then compared the source-language CIQOL-35 instrument with its back translation. Sections of the back translation that were discrepant to the source were documented in the same spreadsheet using color coding for easy identification of sections that were problematic and/or required review.

### Step 4. Committee Review

#### Summary of Best Practices

According to Hall et al. (2018b), this step recommends appointing a multi-disciplinary committee that includes linguistic and healthcare expertise to review the translation steps including the forward and back-translations and review and solve the problematic sections identified in Step 3.

#### Case Study: Translation of the Cochlear Implant Quality of Life-35 Instrument From English to French

The committee review included the three people who translated the PROM in Steps 2–3, the person who completed the reconciliation of the two forward translations and the comparison of the source-language instrument with the back translation, and two bilingual hearing care professionals. All committee members were provided the spreadsheet documenting all steps before the review. During the review meeting, all problematic sections identified in Step 3 as well as any other sections that committee members deemed relevant to discuss were reviewed. As much as possible, consensus was sought on preferable translation. Discussions and reasons underlying translation choices were documented in the spreadsheet. The optimal translation and cultural adaptation of English expressions and figures of speech that do not have direct equivalents in French language/culture such as “crowded environments,” “to socialize,” “social situations,” or “to feel inadequate” generated the most discussion, to ensure semantics were preserved as much as possible whilst creating a culturally appropriate translation. Overall, the translation was at times too literal and benefited from a deeper translation and slight cultural adaptation. During the committee review, any questions raised regarding the original intent of the CIQOL-35 items were noted.

Because significant improvements were suggested to the translation, four additional steps not mentioned in Hall et al. (2018b) were taken. First, a native French speaker naive to the CIQOL-35 reviewed the latest French translation to ensure natural language. As a result, minor changes such as in the choice of prepositions and adverbs were implemented. Second, questions regarding the original intent of the CIQOL-35 were raised with its developers and the translation was slightly revised accordingly. Third, as some questions were raised about the appropriateness of the translation for the intended audience, a native French speaker with hearing loss naive to the CIQOL-35 reviewed the latest French translation to ensure appropriateness. As a result, one minor change to one item was made. Fourth, because significant changes were made since the previous back translation, another back translation was performed on the latest version of the translation. This back translation was completed by a professional translator not involved in previous steps. The same process of comparison and reconciliation described earlier was completed to result in a French translation of the CIQOL-35 ready for field testing (i.e., validation). Whilst it could be argued that the extra steps taken are evidence of suboptimal translation practices, it is believed that they reflect an attention to detail that PROM translation deserves. Table 3 is an excerpt of the reconciliation report: it presents the 26 columns documenting the different steps of the translation for one of the CIQOL-35 items. This reconciliation report was adapted from Hall et al. (2018b) supplemental file 3.

**TABLE 3:** Reconciliation report and how it was adapted from Hall et al. (2018b) supplemental file 3 for the purposes of the present translation project.

Step (Hall et al., 2018b)	Column in reconciliation report	Title Description	Example
2. Forward translation	1	**Descriptor of CIQOL section** Identifier of the section of the SL CIQOL	Item 3
	2	**SL English CIQOL (United States 2019)** Section of the SL CIQOL	If I am interested, I will join family or friends for a social event
	3	**FT #1 French** Made by native speaker professional translator; With red font used to flag discrepancies identified during reconciliation (7–8 below)	Je n’hésite pas à participer à des réunions d’amis ou à des réunions de famille si j’en ai envie.
	4	**Scoring of FT difficulty by professional translator** [0–10], where 0 is extremely easy and 10 is extremely hard	7
	5	**FT #2 French** Made by native speaker health professional; With red font used to flag discrepancies identified during reconciliation (7–8 below)	Si un événement social m’intéresse, j’y participe avec ma famille ou mes amis
	6	**Scoring of FT difficulty by health professional** [0–10], where 0 is extremely easy and 10 is extremely hard (same as 4 above)	4
	7	**Reconciliation of FT** Combination of the two independent FTs (3 and 5 above), with dedicated effort spent on the sections rated as relatively more difficult to translate compared to other sections (4 and 6 above)	Si un événement social avec ma famille ou mes amis m’intéresse, j’y participe.
	8	**Reconciliation Reasoning** Where red font flagged discrepancies between the two FTs, reasons for selecting one translation over another	“N’hésite pas” and “j’ai envie” are less neutral in meaning
3. Back translation	9	**BT** Made by native speaker professional translator naive to the SL CIQOL	If I am interested in a family gathering or social event, I participate.
	10	**SL-BT discrepancy classification** A: Perfect semantic equivalence and good literal and semantic parallels B: Satisfactory semantic equivalence, but have used one or two different words C: Preserves the meaning of the SL, but without satisfactory semantic equivalence D: No agreement	B
	11	**Reconciliation Reasoning** Reasons for adjusting the FT based on the input of the BT, if relevant	Form different but meaning mostly preserved
	12	**Updated FT** With sections in red requiring input from SL developers and/or committee review	Si un événement social m’intéresse, j’y participe avec ma famille ou mes amis.
	13	**Questions for the SL developers and their comments** To clarify original meaning of SL CIQOL sections, with comments identified by SL initials	Clarify meaning of SL item to identify best translation TRM: SL meaning mostly preserved in BT JRD: Slight difference, SL meaning is “going/joining with family and friends to a social event”
	14	**Updated FT** After input from SL developers, before committee review, with sections in red requiring input from committee review	Si un événement social m’intéresse, j’y participe avec ma famille ou mes amis.
4. Committee Review	15	**Questions for committee review** Divided into background and question With reference to 14 above for context	Background: Let’s check the FT against the SL given the BT-SL discrepancies Question: Ok with “Si un événement social m’intéresse, j’y participe avec ma famille ou mes amis.”?
	16	**Comments from committee review** With comments identified by committee review participant initials	ACB: Si un événement social m’intéresse, j’y rejoins ma famille ou mes amis. KJ/EF: Supports FT offered in item 5 above EF: Je n’hésite pas à participer. si j’en ai envie.
x. Additional steps not mentioned in Hall et al. (2018b)	17	**Review from native French speaker naive to the SL CIQOL** To ensure natural language	N/A: no comment provided on this item
	18	**Updated FT** On the basis of 16–17 above	Je n’hésite pas à participer à un événement social avec des amis ou de la famille si j’en ai envie.
	19	**Questions for the SL developers and their comments** To clarify original meaning of SL CIQOL sections, with comments identified by SL initials	N/A: no comment provided on this item
	20	**Updated FT** After input from SL developers,	Je n’hésite pas à participer à un événement social avec des amis ou de la famille si j’en ai envie.
	21	**Review from native French speaker with hearing impairment naive to the SL CIQOL** To ensure appropriateness	N/A: no comment provided on this item
	22	**Updated FT** On the basis of 21 above	Je n’hésite pas à participer à un événement social avec des amis ou de la famille si j’en ai envie.
	23	**BT** Performed by professional translator naive to the translation process so far	I do not hesitate to participate in a social event with friends or family if I want to.
	24	**SL-BT discrepancy classification** A: Perfect semantic equivalence and good literal and semantic parallels B: Satisfactory semantic equivalence, but have used one or two different words C: Preserves the meaning of the SL, but without satisfactory semantic equivalence D: No agreement (same as 10 above)	B
	25	**Reconciliation Reasoning** Reasons for adjusting the FT based on the input of the BT, if relevant	Wording slightly different but meaning fully preserved
	26	**Updated FT** FT ready for field test	Je n’hésite pas à participer à un événement social avec des amis ou de la famille si j’en ai envie.

*We also added the date and person responsible for each step/column in the reconciliation report. BT, back translation; FT, forward translation; SL, source language.*

### Step 5. Field Testing

#### Summary of Best Practices

According to Hall et al. (2018b), this step involves testing the translated PROM on a small group of people drawn from the intended audience. The aim of the field test is to ensure the intended audience understands the translation and finds it acceptable. The field test also aims to ensure that the translation is equivalent to the source-language PROM. Qualitative and/or quantitative methods can be used to reach these aims. Two types of equivalence are typically sought: equivalence of meaning, also called semantic or conceptual equivalence, obtained through careful translation process and qualitative field test, and equivalence of measurement, obtained through careful development and quantitative field test, with CTT (internal consistency) or with IRT (differential item functioning; Petersen et al., 2003; Eremenco et al., 2005), as described earlier. Hall et al. (2018b) state that field testing “is important before proceeding to a wider evaluation of its psychometric properties or before using the translation in real clinical research” (p. 171).

#### Case Study: Translation of the Cochlear Implant Quality of Life-35 Instrument From English to French

It is planned to conduct field testing in two parts. The first part will involve cognitive interviews, a qualitative research method, in a purposely selected and small sample of French-speaking adult patients with CIs. Data collection in close collaboration with two French-speaking CI centers located on two continents (Africa and Europe) have been initiated. This geographical diversity will help determine whether the French-language CIQOL-35 can be used in different French-speaking communities around the world. An interview guide in French has been prepared to facilitate the cognitive interviews. The interview guide queries the respondents on their understanding of the items and the response options and of the cognitive processes engaged when mapping their experiences to the response options. The interview guide also asks whether any item is unsuitable or offensive in the culture of the person completing the PROM. The goal is for the PROM to be a valid representation of the lived experiences of the patients whilst not being cognitively taxing and being acceptable. The responses to the cognitive interview questions will be noted by the interviewers and will be analyzed using content analysis (Graneheim and Lundman, 2004). The second part of the field testing will involve pilot testing in a randomly selected sample of French-speaking adult patients with CIs, without the same emphasis on geographical diversity. Their scores will be summarized using descriptive statistics.

### Step 6. Review and Translation Finalization

#### Summary of Best Practices

According to Hall et al. (2018b), this final step includes the review of the Step 5 results and their incorporation into the final translation, archiving, and dissemination.

#### Case Study: Translation of the Cochlear Implant Quality of Life-35 Instrument From English to French

It is planned to incorporate results of both quantitative and qualitative field testing activities in the final version of the French-language CIQOL-35. It is hoped that the final translation of the PROM will be widely shared free of charge on the Medical University of South Carolina website and disseminated to researchers, clinicians, hearing intervention program developers, and any other people interested in the measurement of CI-related quality of life in adults.

## Anticipated Results and Discussion

It is hoped that this careful translation and cultural adaptation of the CIQOL-35 instrument will lead to a psychometrically sound PROM for French-speaking populations. Ideally, French-speaking adults will find the instrument a relevant and suitable tool to capture the extent to which hearing impairment and hearing interventions impact their CI-related quality of life. Such PROMs are instrumental to quality of care monitoring and improvement.

The translation and cultural adaptation of the CIQOL instruments to eight other languages (Arabic, Danish, German, Hebrew, Malay, Mandarin, Spanish, and Turkish) has been initiated. Cultural adaptation is facilitated by translators living in the location where the translated PROM will be used and by qualitative validation (e.g., with cognitive interviews) to ensure the items are appropriate, understandable, relevant and respectful (i.e., not offensive). In some of the regions in which these languages are spoken, there are no validated speech recognition word/sentence lists. Thus, these PROMs will be heavily relied on to monitor treatment outcomes. The extent of relationships between PROMs needs to continue to be investigated and reported. A related movement is the development of core outcome sets. A core outcome set is an agreed minimum battery of outcome measures to be included in clinical trials. Clinicians and researchers are free to add additional outcome measures, but adherence to a core outcome set ensures that some outcome measures are consistently collected and reported. Core sets have the advantage to allow for cross-trial comparisons and data pooling in meta-analyses. An iterative and multisectoral project led to the World Health Organization’s International Classification of Functioning, Disability and Health Core Sets for hearing loss (ICF Research Branch, 2017). Only a few of the Core Set categories can be measured through physiological and behavioral tests routinely used in clinical and research practice: many more can be evaluated with PROMs. This core set is currently being validated in the population of interest (Karlsson et al., 2021). Core sets are starting to emerge regarding different sub-populations of people with hearing disorders (Hall et al., 2018a; Hill-Feltham et al., 2020; Katiri et al., 2020).

Overall, our experience has taught us that the process of PROM development, translation, and cross-cultural adaptation requires significant time and resources. Therefore, it is best to consider this type of work as a stand-alone project well ahead of time rather than as a quick endeavor when the need for a translation of a PROM becomes apparent.

### Future Trends in Hearing-Related Patient-Reported Outcome Measures

The promises that technologies offer in improving healthcare delivery have been described for decades. The COVID-19 pandemic, and its physical distancing imperative, has accelerated trends toward hybrid hearing healthcare services, combining traditional face-to-face as well as remote care modalities, such as telehealth. Care modalities can be chosen based on patient and context needs. This trend is relevant to PROMs as remote data collection is time efficient and allows the measurement to take place at a time and place that is convenient for the patient, rendering the measure a better reflection of true everyday functioning. Ecological momentary assessment is interesting in a world where ubiquitous technology is increasingly following us, quantifying, tracking, and even pre-empting our behaviors and thoughts. The convergence of PROMs with hearing device usage, acoustic environments, and health and wellbeing data provides a holistic view of a patient’s level of functioning against the context and environment in which they evolve (Timmer et al., 2018). Cloud-based programming of hearing devices also calls for PROMs that are easier and closer to the patient so that replacing face-to-face appointments with remote care does not have to compromise opportunities for outcome measures. Method of administration as well as timing in the course of care can impact on completion and scores. For example, evidence shows that people with hearing impairment may complete PROMs differently when administration is done with pen and paper vs. online (Thorén et al., 2012).

#### Computerized Adaptive Testing

A promising mode of PROM administration is CAT, where an algorithm selects, based on item difficulty and patient responses to previous items, an individualized set of items from a bank of IRT-calibrated items. Items are presented until a predefined measurement precision is reached, or a pre-set maximum number of presented items is reached. CAT increases measurement precision without increasing administration time, thus reducing burden for patients, clinicians, and researchers. Often CATs can provide a similar degree of precision as the full item bank, with completion of far fewer items (Choi et al., 2010; Fries et al., 2014; Pilkonis et al., 2014), and are easily adapted to smartphone or tablet administration. CATs have been developed for each of the CIQOL domains (CIQOL-CAT). Final validation and reliability testing is pending.

#### Patient-Reported Experience Measures

Whilst this manuscript focuses on PROMs, some authors differentiate those from patient-reported experience measures (PREMs) (Kingsley and Patel, 2017). PREMs gather the patient’s perspectives and views of their care experience. Whilst PROMs measure care outcomes, PREMs measure how the patient experienced the care process, for example in terms of communication skills, patient-centeredness, and timeliness. PREMs lead to information central to improve care; they are currently underused within hearing care.

#### Economic Evaluation and Allocation of Scarce Resources

In an era of accountability in healthcare, economic evaluations are increasingly needed to inform the careful allocation of scarce resources. These compare the benefits and costs of several treatment options and use health state values, or utilities, representing people’s preferences for a given health state. The cost per quality-adjusted life year (QALY) or disability-adjusted life year (DALY) and the comparison of incremental cost-effectiveness ratios to thresholds inform the evidence-based prioritization of interventions across health conditions. A recent systematic review identified 117 published economic analyses of hearing healthcare across the continuum of care from prevention and screening to CI and hearing aid provision (Borre et al., 2021). Of those, 62% measured health outcomes in QALYs and 12% in DALYs.

The measurement and valuation of the benefits of medical devices have challenges. First, costs are easier to measure and value than benefits (Thum et al., 2020). Second, generic PROMs are not suitable given limitations. The impact of CI on quality of life in older adults has been measured with the HUI2 and HUI3 (Andries et al., 2021), however EQ-5D lacks construct validity for hearing and HUI3 exhibits ceiling effects, uses “hearing aid” in the item, and measures hearing ability through speech reception only; generic PROMs underestimate the impact of hearing intervention such as CIs (McRackan et al., 2019a). Therefore, it is imperative that PROMs allow for the suitable valuation of hearing intervention benefits.

#### Patient-Reported Outcome Measures in Low-Resource Settings

Patient-reported outcome measures are also of interest in low-resource settings, such as low- and middle-income countries. Their advantages include administration that does not require trained professionals and allows rapid assessment, which makes them interesting as part of monitoring and evaluation of both clinical as well as public health initiatives (Kaspar et al., 2021). Also, PROMs, unlike other forms of outcome measures, do not rely on specialized equipment that requires frequent calibration.

### Clinical Applications of Patient-Reported Outcome Measures

The clinical application of PROMs should be both the start and the end point in PROM development. There is a misconception that if a PROM is carefully developed and translated, it will automatically, or almost magically, be implemented when ready. Careful knowledge translation and implementation science are mandatory for sustainable changes in practice. PROMs improve communication and counseling between professionals and patients (as well as inter-professional communication) regarding the health condition and its impact on quality of life (Greenhalgh et al., 2018). Still, they are underused. Learnings from implementation science can support the successful usage of PROMs to address common implementation barriers such as PROMs inadequately integrated in electronic health record systems, uncertainty about how or why to use PROMs to improve patient care, and clinical workflows that are not conducive, for example due to time pressures (Stover et al., 2020). The successful implementation of PROMs in routine clinical care requires that organizations invest time and resources in the early stages of “designing” the processes for using PROMs (i.e., planning not just which PROMs to use and how to administer them, but also how the data would be used for clinical purposes) and “preparing” an organization and its staff (i.e., getting an organization and its staff ready to use PROMs, particularly persuading clinicians of the validity and value of PROMs, delivering training, and developing electronic systems; Foster et al., 2018).

Selecting the best PROM is paramount. A systematic review concluded that eight criteria should inform PROM selection: appropriateness (match between PROM and specific purpose including research questions if relevant), reliability (reproducibility and internal consistency), validity (whether the PROM measures what it intends to), responsiveness (PROM sensitivity to changes of importance to patients), precision (number and accuracy of distinctions the PROM make), interpretability (how meaningful the PROM scores are), acceptability (how acceptable patients find PROM completion), and feasibility (extent of effort, burden and disruption to staff and clinical care arising from using the PROM; Fitzpatrick et al., 1998). The target patient group, the treatment, and the outcome of the PROM should match the clinical needs. Prior use in groups of similar patients is particularly helpful, and a pilot of the PROM implementation questionnaire can help identify any potential barriers. A short and relevant instrument that is future proof is more likely to be sustainably implemented. An easy, license-free online access to the latest version of any PROM is also conducive to implementation. Of course, if the PROM has been translated, the quality of this process should be documented. The timing and the method of administration should also be carefully considered (Bernstein et al., 2020).

Furthermore, the hearing community needs to reach consensus on the most important outcome domains and the core set of measures to assess these domains in a consistent and therefore comparable fashion in clinical research, clinical trials, and in the monitoring of the impact of hearing health policies. Current minimum reporting standards for adult CI do not include PROMs (Adunka et al., 2018). The hearing community can learn from other fields where core sets of measures of treatment effects include PROMs. Medical device regulators worldwide are also increasingly asking for the systematic collection and reporting of PROMs during the entire product lifecycle.

### Summary and Conclusion

This case study centered around the CIQOL-35 Profile instrument measuring functional abilities and quality of life in adults with hearing impairment, showed that the development of PROMs should be driven by real-world needs. The development of PROMs must start with clinical need and must ensure active involvement of important stakeholders at all stages. This is the case of the CIQOL suite of instruments, which benefited from a systematic literature review and focus groups with patients, who are experts in lived experiences of hearing impairment. The mixed methods used in the development of the CIQOL suite of instruments enhance and expand their potential applications.

This paper concludes with four suggestions for people embarking on similar endeavors:

1. Think clinical applications first, in terms of populations, concepts to be measured, etc.
2. Start with performing a literature review and inviting patient perspectives, because there is no need to reinvent the wheel and because patients are the experts into the lived experiences of a health condition.
3. Adhere to published standards of both development as well as translation and cultural adaptation, because there exists a large body of psychometric science to draw from.
4. Aim for PROMs that will stand the test of time, for example in terms of content and modes of administration.

## Data Availability Statement

The original contributions presented in the study are included in the article/supplementary material, further inquiries can be directed to the corresponding author/s.

## Author Contributions

All authors listed have made a substantial, direct, and intellectual contribution to the work, and approved it for publication.

## Conflict of Interest

AL-L is employed by Oticon Medical A/S, part of Demant A/S, a hearing healthcare company. TM is an educational consultant with Stryker Corporation and on the medical advisory board of Envoy Medical. The remaining authors declares that the research was conducted in the absence of any further commercial or financial relationships that could be construed as a potential conflict of interest. The authors declare that this study received funding from Oticon Medical (ALL) and NIH/NIDCD K23 DC016911 (TRM). The funders were not involved in the study design, collection, analysis, interpretation of data, the writing of this article or the decision to submit it for publication.

## Publisher’s Note

All claims expressed in this article are solely those of the authors and do not necessarily represent those of their affiliated organizations, or those of the publisher, the editors and the reviewers. Any product that may be evaluated in this article, or claim that may be made by its manufacturer, is not guaranteed or endorsed by the publisher.

## Footnotes

1
https://hub.lexile.com/analyzer
